# Repositioning intellectual disability in the ethics of digital mental health technologies

**DOI:** 10.3389/fpsyt.2025.1691940

**Published:** 2025-10-21

**Authors:** Anithamol Babu, Akhil P. Joseph

**Affiliations:** ^1^ School of Social Work, Marian College Kuttikkanam Autonomous, Kuttikkanam, India; ^2^ School of Social Work, Tata Institute of Social Sciences, Guwahati, India

**Keywords:** intellectual disability, digital mental health, cognitive justice, algorithmic exclusion, AI ethics, participatory design, disability justice, epistemic inclusion

The rapid expansion of Artificial Intelligence (AI) and digital platforms in mental health care has introduced promising tools for screening, triage, and psychoeducation. Yet, for individuals with intellectual disabilities (ID) - a heterogeneous group encompassing varying levels of cognitive functioning, communication abilities, and support needs- this technological shift has intensified rather than ameliorated pre-existing inequities. Intellectual disability includes individuals with mild to profound impairments in cognitive processing, often intersecting with limitations in adaptive behavior, verbal communication, and decision-making autonomy (1). These variations shape how individuals interact with and benefit from digital mental health systems. Despite growing discourse around inclusive design, the near-total absence of individuals with ID in digital mental health research and system architecture remains a paradigmatic failure (2). This exclusion is not a function of technological incapacity but stems from entrenched epistemological and clinical assumptions that erase cognitive variance as a legitimate form of mental health subjectivity. Addressing this omission demands a foundational reimagining of how digital mental health systems conceptualize intelligence, usability, and therapeutic engagement.

The prevailing dominance of the psychiatric-medical model in digital mental health development reinforces a reductive, pathologizing approach to intellectual disability (3, 4). Rooted in the notion of individual deficiency, this model underpins many algorithmic frameworks that normalize neurotypical patterns of cognition and emotional regulation. Consequently, digital interventions—from AI chatbots and self-screening tools to emotion-sensing technologies—often rely on decision trees and behavioral templates that render individuals with ID algorithmically invisible (5–7). These systems lack responsiveness to cognitive and communicative differences and often misinterpret divergent behaviors as dysfunction. While some efforts in digital mental health attempt to integrate social determinants and participatory frameworks, these remain peripheral to dominant logics that continue to prioritize normative constructs of cognition. This exclusion is embedded not only in technical models but in design infrastructures that assume linear logic, rational agency, and verbal fluency—traits aligned with neurotypical cognition. Technologies driven by natural language processing, adaptive learning, and emotion recognition frequently misconstrue non-linear cognition, atypical affect, and symbolic expression as errors. These systems reflect embedded norms of legibility and intelligibility, delegitimizing alternative ways of knowing, expressing, and relating. This aligns with broader critiques of epistemic oppression in disability scholarship, which identify academic gatekeeping and communicative inaccessibility as mechanisms of exclusion (8).

AI’s potential to enhance communication, education, and independence for people with ID is often acknowledged—particularly through personalized support, early diagnosis, and adaptive environments. However, these narratives are frequently framed within skill-based discourses that treat disability as a variable for technical adaptation rather than a standpoint for epistemological and design reconfiguration. Algorithmic fairness frameworks dominate equity discourses, yet intellectual disability is rarely included in fairness metrics or audit protocols, risking systems that reproduce structural exclusion beneath a veneer of inclusivity (9, 10). One critical vector of exclusion lies in the construction of training datasets. Mental health datasets often originate from normatively defined populations, systematically excluding individuals with ID. This leads to algorithmic misclassification or total omission. In high-stakes domains such as suicide risk prediction or digital phenotyping, such exclusion results in erroneous assessments or denial of services. These gaps reflect a self-reinforcing feedback loop: exclusion from data produces exclusion from care.

Standard research methodologies in digital mental health often preclude inclusion by relying on tools for consent, symptom tracking, and user feedback that presume verbal fluency and linear cognition, thereby excluding individuals who communicate symbolically, non-verbally, or with cognitive divergence. Quantitative methods value standardization over accessibility, while qualitative methods rarely adjust to include co-production with individuals with ID. Ethical review boards frequently label these individuals as “inherently vulnerable,” enacting protective exclusions that erase agency and perpetuate epistemic injustice. The medical model’s entrenchment also persists in mainstream AI ethics and policy frameworks. Instruments like the EU AI Act and UNESCO’s Ethical AI Recommendations prioritize transparency and risk management but omit mandates for cognitive diversity or participatory governance (11–13). As such, cognitive justice—defined here as the equitable recognition and integration of diverse ways of knowing—remains largely absent from regulatory landscapes.

Alternative frameworks offer critical correctives. Disability justice emphasizes intersectional leadership and challenges exclusionary knowledge production. Participatory and disability-led design promotes co-creation through accessible modalities. Disability data justice views data infrastructures as sites of struggle, advocating for community-defined priorities. The anti-ableism framework urges developers to recognize disability as a central identity category. DIKWP’s semantic justice model bridges cognitive diversity and legal reasoning, advancing inclusive algorithmic jurisprudence. These frameworks, while promising, remain marginal in practice. Their integration into high-risk AI deployments requires enforceable mandates: cognitive accessibility standards, participatory design protocols, and metrics for epistemic inclusion—defined here as the deliberate integration of diverse cognitive, communicative, and experiential knowledge systems into the full life cycle of digital mental health technologies, from design to governance. Ethics curricula must address cognitive diversity, disability justice, and capability-informed evaluation. Policymaker-academic collaborations with grassroots organizations are essential to institutionalize these approaches.

A comparative analysis of digital mental health platforms highlights the consequences of epistemic exclusion and the emerging potential for inclusive redesign. Many mainstream tools are structured around neurotypical patterns of communication and interaction, which may not align with the cognitive preferences, expressive modes, and interpretive frameworks of users with intellectual disabilities (14–16). However, there is currently no robust empirical data to confirm or refute this misalignment, highlighting a significant gap in our understanding of how digital mental health systems interact with cognitive diversity. This evidence gap complicates our understanding of whether digital mental health tools are therapeutically effective, accessible, or even safe for individuals with intellectual disabilities. Broader accessibility concerns also persist, as many mental health apps remain unevaluated for use by disabled populations, despite claims of expanding access (17). These concerns collectively underscore the need for a unified participatory design approach—one that centers people with intellectual disabilities throughout development and evaluation, and incorporates their lived experiences, communicative strategies, and cognitive capacities across age groups and clinical settings (16, 18, 19).

Emotion AI systems misclassify atypical affect; accessible games overwhelm users with cognitive processing limitations; symbolic or minimally verbal users are excluded from platforms like Guremintza and Wikibase (20–22). Stress-regulation apps often presume autonomous navigation and structured comprehension. Encouragingly, efforts like iterative playtesting, pictorial UIs, semantic navigation tools, and iconographic dashboards reveal the feasibility of inclusive co-design. Microsoft’s AI for Accessibility initiative and the UK-based AbleChat pilot signal early but promising shifts from symbolic inclusion to structural participation (23, 24).

However, in low- and middle-income countries (LMICs), the stakes are magnified. Imported AI tools built on Western cognitive norms often invalidate local caregiving practices, knowledge systems, and expressions of distress. Without contextual adaptation, such tools operate as instruments of digital colonialism, perpetuating cognitive and cultural hierarchies. For example, in several rural areas across South Asia and Sub-Saharan Africa, digital mental health apps are accessed through shared mobile phones with limited data connectivity, often controlled by caregivers or community health workers. These conditions not only limit individual autonomy but also challenge the feasibility of consistent therapeutic engagement without offline functionality or adaptive, low-bandwidth interfaces. To counter these realities, coordinated and context-sensitive reforms are necessary. Procurement systems must mandate cognitive accessibility audits and integrate local disability organizations in platform evaluations. Funding bodies should require multi-phase co-design protocols, enabling the full participation of individuals with ID through Easy Read materials, pictorial tools, and supported decision-making. Governance bodies must ensure representation through permanent ID-affiliated seats. Implementation strategies must address infrastructural disparities with offline functionality, shared-device access, and community-based digital facilitation. Academic institutions should lead the formation of interdisciplinary hubs focused on disability ethics, inclusive AI design, and participatory research to ensure sustained innovation.

This article calls for interdisciplinary scholars, technologists, clinicians, disability advocates, and policymakers to radically reimagine digital mental health as a site for epistemic reconstruction rather than retrofitted inclusion. It argues that intellectual disability must be elevated from the periphery of accessibility compliance to the center of digital mental health design, governance, and evaluation. While recognizing the limitations inherent in an opinion-based article—including the absence of new empirical data and the reliance on selective case illustrations—this piece aims to lay a conceptual foundation for future inquiry and participatory research agendas. As an actionable starting point, we propose a “minimum standards” checklist for digital mental health systems: (1) mandatory cognitive accessibility audits during procurement; (2) inclusion of individuals with ID in training datasets; (3) deployment of supported decision-making tools and Easy Read formats; (4) co-design protocols with ID stakeholders; and (5) governance structures that guarantee representation of individuals with intellectual disabilities through permanent advisory roles. This requires moving beyond symbolic gestures and implementing a bold, empirically driven research agenda that challenges entrenched norms, disrupts dominant frameworks, and embeds cognitive justice as a foundational principle across the life cycle of technological innovation. The field must commit to structurally embedding the lived experiences, communication modes, and relational epistemologies of people with intellectual disabilities into the infrastructures, algorithms, and oversight systems that define AI-driven mental health care. This transformative agenda should be anchored in sustained collaboration across psychiatry, cognitive disability studies, human-computer interaction, AI and machine learning, bioethics, implementation science, and health economics to co-create systems not merely inclusive of, but co-authored by, people with intellectual disabilities. Priority areas for research include the development of representative data infrastructures that capture cognitive variance; the institutionalization of cognitive-justice evaluation metrics that supplement conventional AI benchmarks; the prototyping and trialing of inclusive interfaces designed through multi-site, adaptive methodologies; the reform of governance structures to ensure representation, accountability, and accessibility; and the contextual localization of digital interventions in low- and middle-income countries, where infrastructural and epistemic inequities compound. If executed with rigor, transparency, and disability-led co-production, this research program can dismantle the prevailing logic of normative accommodation and replace it with a paradigm of structural co-creation—yielding digital mental health systems that are not only technically robust and clinically effective, but epistemically inclusive and socially transformative.
